# Focusing the Spotlight on Lack of Access to Health Information

**DOI:** 10.1371/journal.pmed.1001438

**Published:** 2013-04-30

**Authors:** The PLoS Medicine Editors

## Abstract

In their April Editorial, the *PLOS Medicine* Editors reflect on the critical need for access to high-quality health information across the globe and on a recent analysis suggesting that governments have a legal responsibility to ensure access to health information for their citizens and health workers.

“In the 21st century, knowledge is the key element to improving health. In the same way that people need clean, clear water, they have a right to clean, clear knowledge” [1]. This is how Sir Muir Gray, Director of the UK's National Health Service (NHS) National Knowledge Service, describes the importance of health knowledge. Knowledge underpins every medical advance, every intervention, and every clinical decision. However, access to reliable health information for even the most basic health needs remains elusive for much of the world's population.

Access to reliable health information remains a problem even in settings where clean water is taken for granted. Despite the recognition of the importance of evidence-based health information, the problems of publication bias [2], missing trial data [3], influence from commercial organizations [4], and distortion of study implications [5] are well known and continue to haunt medical science and the information available to health workers and the general public. In addition to these challenges to the medical evidence, the process of translating available knowledge into appropriate action is a complex and ongoing endeavor [6].

It is in the poorest settings where basic health information may prove most valuable. For example, postpartum hemorrhage (PPH) is a leading cause of maternal death worldwide; yet despite being recommended by the WHO and other professional bodies, active management of the third stage of labor to prevent PPH was found to be correctly used in only 0.5% to 32% of observed deliveries in seven developing countries [7]. Worryingly, six of the seven countries were found to have multiple guidelines and conflicting recommendations for active management of the third stage of labor [7]. While lack of reliable information may well be a symptom of a weak health system in the most extreme cases, it can be the result of misinformation. It has been estimated that more than 330,000 lives were lost between 2000 and 2005 because the then-government of South Africa questioned whether HIV was the cause of AIDS, and they failed to implement a feasible and timely antiretroviral treatment program [8].

Medical journals remain a key part of the knowledge translation process, almost exclusively dealing with the final stages of knowledge creation (primary research), distillation (systematic reviews and guidelines), and commentary (editorializing and contextualizing by experts) via peer review and finally dissemination. Although making research openly available to be both read and reused is an essential step toward a vision of wider access to healthcare knowledge, disseminating information on its own is not enough to ensure evidence is used in decision-making [9]. In many settings it is access to secondary reference and educational materials based on the best available evidence that is severely lacking yet probably more crucial for clinical practice than the most recent observational study or clinical trial findings.

Organizations such as the WHO among others play an important role in providing reliable healthcare information. However, in low- and middle-income countries, such information is often not available where it is needed, or the information is not usable because it is in the wrong language or because it does not match the context or level of education of the healthcare provider.

In a recently published white paper, Neil Pakenham-Walsh and Molly Land argue that, because access to health information is a key determinant to the human right to the highest attainable standard of health, governments have a legal responsibility under international human rights law to provide access to healthcare information to citizens and health workers [10]. That is not to say that governments are required to generate this information, but they must ensure its availability and an enabling policy environment that does not hinder access to health information. States should provide access to information about health services and health policy so that a country's citizens can access those services when needed and the educational health needs of both the general population and health workers are met.

If governments are legally obliged to enable access to reliable health information, what can be done to ensure that they do so? It is unlikely that governments will be held legally responsible for not ensuring that health information is available to their citizens and health workers, and a legal approach would be inappropriate in most cases. Furthermore, it is unrealistic to expect governments to react quickly to calls for change. However, by placing access to reliable health information into the broader human rights framework it may be possible to benefit from the momentum already generated by human rights organizations.

One model that has been effectively used by organizations such as Human Rights Watch (www.hrw.org) and Amnesty International (www.amnesty.org) to promote change is holding up a light to practices of governments, raising awareness of where they fail to meet their responsibilities. Healthcare Information for All by 2015 (HIFA2015) has taken this approach by setting up a campaign called HIFA-Watch (http://www.hifa2015.org/hifa-watch/). The campaign aims to highlight positive examples, such as recent legislation in Pakistan to ensure that commercial companies cannot claim that formula milk is a substitute for breast milk [11], as well as negative examples of government practices, such as countries that do not legally require pictorial warnings on tobacco products [12]. Of course, a webpage alone will not ensure change, and research into the practices of individual governments and sustained momentum are needed in order for the campaign to be a success.

The challenge of improving healthcare information in countries with meager resources will require more than just highlighting insufficiencies. Access to health information is a key component of a strong health system, but to be effective it requires evaluation and synthesis of evidence, translation of evidence into educational materials, and implementation and dissemination. Health information is one key component of the complex task of improving weak health systems, along with cooperation, political will, and funding.

## Abbreviations

NHS: National Health Service
PPH: postpartum hemorrhage
